# Impact of endobiliary radiofrequency ablation on survival of patients with unresectable cholangiocarcinoma: a narrative review

**DOI:** 10.3389/fonc.2023.1077794

**Published:** 2023-05-30

**Authors:** Elena Di Girolamo, Andrea Belli, Alessandro Ottaiano, Vincenza Granata, Valentina Borzillo, Luca Tarotto, Fabiana Tatangelo, Raffaele Palaia, Corrado Civiletti, Mauro Piccirillo, Valentina D’Angelo, Francesco Fiore, Pietro Marone, Guglielmo Nasti, Francesco Izzo, Mario de Bellis

**Affiliations:** ^1^ Division of Gastroenterology and Gastrointestinal Endoscopy. Istituto Nazionale Tumori, IRCCS, Fondazione G. Pascale, Naples, Italy; ^2^ Division of Hepatobiliary Surgery. Istituto Nazionale Tumori, IRCCS, Fondazione G. Pascale, Naples, Italy; ^3^ Unit for Innovative Therapies of Abdominal Metastastes. Istituto Nazionale Tumori, IRCCS, Fondazione G. Pascale, Naples, Italy; ^4^ Division of Radiology. Istituto Nazionale Tumori, IRCCS, Fondazione G. Pascale, Naples, Italy; ^5^ Division of Radiotherapy. Istituto Nazionale Tumori, IRCCS, Fondazione G. Pascale, Naples, Italy; ^6^ Division of Interventional Radiology. Istituto Nazionale Tumori, IRCCS, Fondazione G. Pascale, Naples, Italy; ^7^ Division of Anatomic Pathology and Cytopathology. Istituto Nazionale Tumori, IRCCS, Fondazione G. Pascale, Naples, Italy; ^8^ Gastropancreatic Surgical Unit. Istituto Nazionale Tumori, IRCCS, Fondazione G. Pascale, Naples, Italy

**Keywords:** cholangiocarcinoma, malignant biliary strictures, endobiliary radiofrequency ablation, ERCP, PTC, biliary drainage, biliary stent patency, overall survival

## Abstract

Cholangiocarcinoma (CCA) is a rare cancer originating from the biliary epithelium and accounts for about 3% of all gastrointestinal malignancies. Unfortunately, the majority of patients are not eligible for surgical resection at the time of diagnosis, because of the locally advanced stage or metastatic disease. The overall survival time of unresectable CCA is generally less than 1 year, despite current chemotherapy regimens. Biliary drainage is often required as a palliative treatment for patients with unresectable CCA. Recurrent jaundice and cholangitis tend to occur because of reobstruction of the biliary stents. This not only jeopardizes the efficacy of chemotherapy, but also causes significant morbidity and mortality. Effective control of tumor growth is crucial for prolonging stent patency and consequently patient survival. Recently, endobiliary radiofrequency ablation (ERFA) has been experimented as a treatment modality to reduce tumor mass, and delay tumor growth, extending stent patency. Ablation is accomplished by means of high-frequency alternating current which is released from the active electrode of an endobiliary probe placed in a biliary stricture. It has been shown that tumor necrosis releases intracellular particles which are highly immunogenic and activate antigen-presenting cells, enhancing local immunity directed against the tumor. This immunogenic response could potentially enhance tumor suppression and be responsible for improved survival of patients with unresectable CCA who undergo ERFA. Several studies have demonstrated that ERFA is associated with an increased median survival of approximately 6 months in patients with unresectable CCA. Furthermore, recent data support the hypothesis that ERFA could ameliorate the efficacy of chemotherapy administered to patients with unresectable CCA, without increasing the risk of complications. This narrative review discusses the results of the studies published in recent years and focuses on the impact that ERFA could have on overall survival of patients with unresectable cholangiocarcinoma.

## Introduction

Cholangiocarcinoma (CCA) is a rare cancer originating from the biliary epithelium and accounts for about 3% of all gastrointestinal malignancies (1, 2). The tumor is classified as intrahepatic, perihilar and distal, according to its anatomical location (1–3). Perihilar tumors represent 50-60% of all cholangiocarcinomas, intrahepatic CCA accounts for 10-20% of cases and extrahepatic cancers involving the main bile duct are diagnosed in 20-30% of patients (3, 4). Surgery offers the best outcome, but the majority (approximately 70%) of patients are not eligible for surgical resection at the time of diagnosis, because of the locally advanced stage or metastatic disease (2–4). The survival time of patients with unresectable CCA undergoing chemotherapy is generally less than 1 year (10.6-11.7 months), while best supportive care is associated with a median overall survival of 5 (2.8-7.7) months (2–4).

Since the majority of patients with unresectable CCA present with malignant biliary obstruction, biliary drainage is a crucial palliative treatment for patients with hilar or distal CCA. This can be obtained either by means of ERCP (Endoscopic Retrograde ColangioPancreatography) or PTC (Percutaneous Transhepatic Colangiography), placing one or more biliary stents (plastic or metal) which relieve jaundice, without changing patients prognosis (5, 6). Unfortunately, recurrent jaundice and cholangitis tend to occur because of reobstruction of the biliary stents due to tumor growth, despite the use of self expandable metals stents (SEMS), which have replaced plastic biliary stents in clinical practice to reduce the occurrence of recurrent jaundice (7, 8). This not only jeopardizes the efficacy of chemotherapy, but also causes significant morbidity and mortality (3, 4). Effective control of tumor growth is crucial for prolonging stent patency and consequently patient survival.

Recently, endobiliary radiofrequency ablation (ERFA) has been experimented as a treatment modality to reduce the tumor mass and delay tumor growth, extending stent patency (9–12). Several studies have demonstrated that ERFA is associated with an increased median survival of approximately 6 months in patients with CCA, without increasing the risk of complications (13–19). However, the improved overall survival could be simply secondary to the effect of ERFA on stent patency, which is usually prolonged by approximately 2 months (20–23). Both the prolonged patency of biliary stents and the delayed tumor growth could be strictly connected and allow a prompt recovery with prolonged jaundice free status, which avoids discontinuation of chemotherapy (9–12, 16).

This narrative review summarizes the results of the studies published in recent years and focuses on the impact that ERFA could have on the overall survival of patients with unresectable cholangiocarcinoma.

## Overview of endobiliary therapy for unresectable cholangiocarcinoma

Endobiliary therapy of the tumor complementing chemotherapy for treatment of patients with unresectable CCA is appealing and it has been evaluated in clinical practice. The majority of patients with unresectable CCA require biliary drainage because of obstructive jaundice. Biliary stenting improves the quality of life but does not extend overall survival of these patients (18). At the same time of biliary drainage, endobiliary locoregional therapy can be administered and the combination of chemotherapy and endobiliary therapy has shown to improve the overall survival and the quality of life in patients with unresectable CCA (9, 24–26). It seems that local control of the tumor growth is crucial and this could be achieved by using different ablative techniques. These can be extrabiliary, like irreversible electroporation (IRE), or endobiliary such as intraluminal brachytherapy (ILBT), photodynamic therapy (PDT), and radiofrequency ablation (RFA) (9, 24–29).

IRE is a non-thermal tumor ablation technique which is mainly indicated for the treatment of locally advanced pancreatic cancer (27). IRE generates high-voltage electric current which induces cell apoptosis, because it alters the permeability of the cell membrane, without damaging the surrounding structures (27–29). Therefore, IRE can be used safely for the treatment of lesions near vascular and biliary vessels (30). Based on these findings, IRE has been used for the treatment of patients with unresectable CCA resulting in prolonged biliary decompression and improvement in both quality of life and overall survival (28, 29). The main limitation of IRE is related to the technique itself which requires surgery (open VS laparoscopic) or percutaneous approach, always performed under ultrasound guidance (27–29). ILBT requires the insertion of iridium-192 (192Ir) or iodine-125 (125I) seeds contained in an impregnated wire which is advanced into the lumen of a nasobiliary tube or an external biliary catheter previously placed at the time of ERCP or PTC, respectively (24, 26). The radioactive seeds are placed inside the biliary stricture under fluoroscopic guidance using the markers present on the wire and high dose radiation (10-20 Gy) is locally delivered reducing the tumor mass, as well as controlling its growth by means of DNA damage, inhibition of cellular replication, and induction of tumor cells apoptosis (9, 24–26). Contiguity of the radiation source to the tumor allows the delivery of a higher dose of radiation, with less adverse effects on the surrounding structures (25). The efficacy and safety of ILBT has been evaluated in several heterogeneous small clinical studies, whose results do not allow to draw final conclusions on its effect in prolonging overall patient survival and stent patency (24, 26). An increased overall survival of the patients has been reported after ILBT in combination with external beam radiation therapy with or without chemotherapy (9, 24–26). The complexity of the procedure, the logistic problems of managing the radioactive material properly and some delayed serious adverse events (duodenal stenosis, gastrointestinal bleeding and hemobilia) have limited the use of ILBT in clinical practice (9, 24, 26).

Endobiliary PDT requires the administration of an intravenous photosensitizing agent (porfimer sodium) which concentrates in malignant biliary cells and is activated by a laser light of a specific wavelength delivered by a laser fiber placed into the biliary tree at the level of the stricture by means of ERCP or PTC (24). Subsequent generation of radical oxygen species with photoperoxidation of cellular membranes leads to apoptosis and necrosis of the neoplastic tissue which is also favored by inflammatory and antiangiogenic pathways locally activated by PDT (9, 24–26, 31). Moreover, the laser light refracting within the bile is transmitted through the biliary system and allows PDT to treat peripheral and unreachable lesions (24). After PDT, endoscopic biliary stenting is required because of tissue inflammation and edema. Plastic stents are preferred to metals stent because they allow repetition of PDT every 2-3 months at the time of stent exchange. However, there is no standardized protocol for endobiliary PDT regarding the number of sessions, interval between sessions, and bilateral vs unilateral endobiliary therapy. Numerous published studies, including several meta-analyses, reported a significant improvement of overall patient survival, and prolonged stent patency after endobiliary PDT (9, 24–26, 31, 32). The association of this ablative technique with chemotherapy has a beneficial effect, resulting in significantly longer overall survival and median progression-free survival of patients undergoing combined therapy (9, 24–26, 31, 32). According to the results of a systematic review and meta-analysis, endobiliary PDT is more effective than ERFA and stenting alone for the treatment of patients affected by unresectable CCA, with significantly prolonged overall patient survival as well as reduced mortality (32). Despite its reported therapeutic efficacy, endobiliary PDT has not become a standard of practice because of its side effects and pitfalls. Increased risk of bacterial cholangitis, liver abscess, and hemobilia are rare, but serious complications (25). Phototoxicity may result in pruritus, diffuse pain, skin erythema, and even blistering which may be prevented by avoiding direct sunlight for 4-6 weeks after PDT (9, 24–26, 31). This significantly affects the quality of life of patients who need to be carefully informed before undergoing PDT, especially if multiple sessions are predicted (9, 24, 26). Other practical downsides are the interval required between the administration of the intravenous photosensitizing agent and the execution of PDT as well as the time needed for each therapeutic session which is approximately 13 minutes (26). Finally, the high cost of each PDT session together with the need of a special laser contributes to the limited application of PDT for the treatment of patients with unresectable CCA (24, 26).

After preliminary experimental studies, in 2011 Steel et al. published the first report of a pilot study which evaluated feasibility, efficacy and safety of ERFA for the treatment of patients with malignant biliary obstruction (MBO) (17). The results of this study stimulated both experimental and clinical research with the objective of introducing ERFA in clinical practice for the management of patients with MBO and especially those with unresectable CCA for whom both ILBT and PDT do not represent the best therapeutic approach (**Tables 1A**, **B**) (9, 26).

**Table 1.A:** Comparison among ILBT, PDT and ERFA (from 24-26).

	ILBT	PDT	ERFA
INDICATIONS	- Perihilar U-CCA - Neoadjuvant therapy associated to chemoradion before liver transplantation in selected patients with CCA	- Perihilar U-CCA - U-CCA Bismuth IV - Neoadjuvant therapy associated to chemoradion before liver transplantation in selected patients with CCA	- Perihilar U-CCA - Distal U-CCA - Adjuvant therapy associated to chemotherapy - Occluded biliary metal stent - Intraductal residual tissue of resected ampullary adenomas
CONTROINDICATIONS	- Poor clinical status (KPS < 50) - Severe liver insufficiency (PT ≤ 40%) - Severe kidney disease (CrCl < 10mL/min)	- Poor clinical status (KPS < 50) - Coagulopathy - Severe liver insufficiency (PT ≤ 40%) - Severe kidney disease (CrCl <10mL/min)	- Poor clinical status (KPS < 50) - Cardiac devices - Coagulopathy - Severe liver insufficiency (PT ≤ 40%) - Severe kidney disease (CrCl <10mL/min)
MECHANISM OF ACTION	- Localized delivery of high-dose radiation - Direct DNA damage - Inhibition of cellular replication - Induction of apoptosis of tumor cells	- Concentration of photosensitizing agent into the cancer cells - Activation of the photosensitizer by exposure to light of a laser fiber - Photoperoxidation of cell membranes - Cancer cells apoptosis	- Heat generation with local T> 50° - Coagulative necrosis and tumor cells death - Release of highly immunogenic intracellular particles - Enhancement of local immunity directed against the tumor
SPECIAL CONSIDERATIONS	- Insertion of iridium-192 (192Ir) or iodine-125 (125I) seeds into the biliary stricture - Need of ribbon or impregnated wire - Nasobiliary tube or external biliary catheter placed at ERCP or PTC - High-dose rate ILBT preferred - Shielded room - Recommended combination of ILBT with external beam radiation - Biliary stenting requiring a second procedure - Relatively expensive: about 14,000 USD $*	- Intravenous administration of photosensitizing agent - Diode laser system - Laser fiber with a cylindrical diffuser at the distal end and specific wavelenght (630 nm) - Delivery of PDT to malignant tissue away from the laser fiber - Recommended endoscopic biliary stenting = plastic vs metal - Repeatable (if plastic stent used) - Expensive: about 50,000 USD $ per PDT session	- Two endobiliary ERFA probe systems: HABIB and ELRA - Dedicated radiofrequency generator (ELRA) - Commercially available electrosurgical generator (HABIB) - Required direct tissue contact to obtain tissue destruction - Recommeded endoscopic biliary stenting = plastic vs metal - Repeatable (if plastic stent used) - Inexpensive: price of an ERFA probe is about 2,300 USD $

PDT, Photodynamic Therapy; ERFA, Endobiliary Radiofrequency Ablation; ILBT, Intraluminal Brachytherapy; U-CCA, Unresctable Cholangiocarcinoma; KPS, Karnofsky Performance Scale; CrCL, Creatinine Clearance; T, temperature; *from the WEB.

**Table 1.B:** Comparison among ILBT, PDT and ERFA (from 24-26).

	ILBT	PDT	ERFA
OUTCOMES compared to stent alone	- Increased Stent Patency - Prolonged Survival	- Prolonged Survival - Longer Stent Patency - Improved KPS	- Improved Survival - Improved Stent Patency
ADVERSE EVENTS	- Cholangitis - Hemobilia - Gastrointesinal Bleeding - Duodenal Stenosis	- Phototoxicity - Abdominal Pain - Cholangitis - Liver abscess - Hemobilia	- Abdominal Pain - Cholangitis - Cholecystitis - Hemobilia - Liver infarction - Intraheptic Pseudoaneurysm
downsides	- Complexity of the procedure - Logistic problems - Challenging management of the radioactive material (handling, storing, devilering) - Radioprotection issues	- 3 day interval between the administration of the intravenous photosensitizing agent and the execution of PDT - Need to avoid direct sunlight for 4-6 weeks after PDT - Long therapeutic sessions (13 minutes)	- Often, more than one session - Low energy settings for ablation of intrahepatic stricture - Impairment of efficacy due to anatomical characteristics - Heat-sink effect

PDT, Photodynamic Therapy; ERFA, Endobiliary Radiofrequency Ablation; ILBT, Intraluminal Brachytherapy; KPS, Karnofsky Performance Scale.

## Endobiliary radiofrequency ablation

ERFA is accomplished by means of a high-frequency alternating current which is released from an active electrode located in the middle portion of an endobiliary probe. This is placed inside the biliary stricture where the subsequent emission of thermal energy causes coagulative necrosis and cellular death when the temperature exceeds 50°C (11, 12). It has been shown that tumor necrosis releases intracellular particles which are highly immunogenic and activate antigen-presenting cells, enhancing local immunity directed against the tumor (33–36).

### Immunogenic response

**Figure 1:**
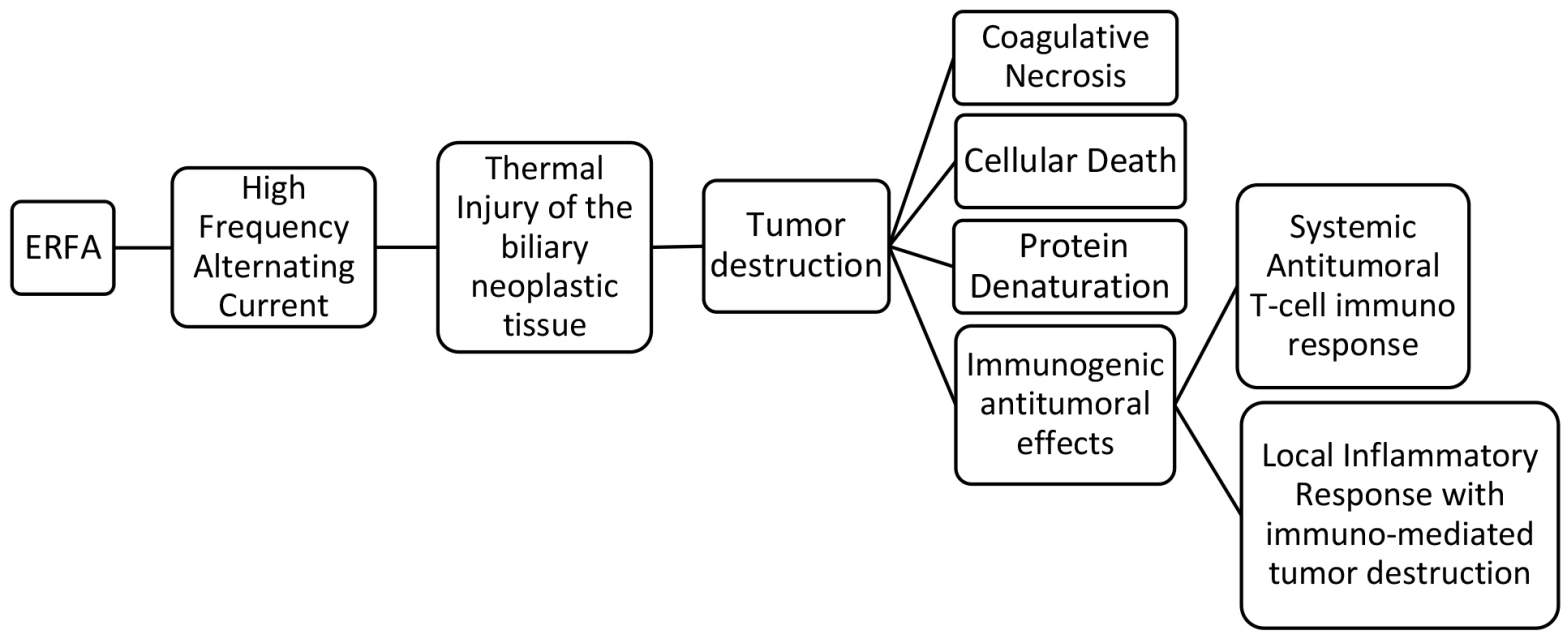
Pathophysiology of Endobiliary Radiofrequency Ablation (ERFA) (modified from 25).

Radiofrequency ablation (RFA) has been shown to induce antigen-presenting cell infiltration and enhance systemic antitumor T-cell immune response as well as tumor regression in hepatocellular carcinoma (36). The tumor necrosis generated could be an antigen source for the immune system and it has been demonstrated that RFA determines a weak but detectable immune response which involves the activation of macrophages and the release of inflammatory cytokines (34). An early increase of cytokine IL6, followed by a delayed elevation of the serum levels of chemokines CXCL11, CXCL5, and CXCL1 was recently demontrasted in patients with pancreatic cancer and cholangiocarcinoma undergoing ERFA (37). However, the systemic immune response detected after ERFA was not specifically related to the endobiliary ablation and it was attributed to a general inflammatory response (37). Most likely, the immunogenic effects of RFA occur at the tumor site where the necrotic neoplastic tissue induces severe inflammation which can determine immune-mediated tumor destruction by neutrophils, macrophages, dendritic cells, natural killer cells together with B and T lymphocytes (33, 37). It has been speculated that the immune-mediated tumor destruction is not triggered by necrotic neoplastic tissue, but it is induced by the immunostimulatory and inflammatory factors present in the sub-vital tissue surrounding the ablated necrotic area (33). This could be particulary true for biliary strictures treated with ERFA where there is no certainty of complete tumor destruction since the width, the depth and the length of the ablation are not foreseeable, as demonstated experimentally (38–43). The local immunogenic response could potentially enhance tumor suppression and be responsible for the improved survival of patients with MBO and unresectable CCA who undergo ERFA (20–23) (**Figure 1**).

### Endobiliary probes

To date, there are two ERFA catheters which have been approved for clinical use (**Table 2**). The HABIB catheter (Boston Scientific, Marlborough, MA, USA) is a power-controlled 8-French bipolar probe, 180 cm long, with two circumferential electrodes placed 8-mm apart on the distal tip of the catheter to achieve an ablation length and depth of 20-25 mm and 3-5 mm, respectively (17, 43, 44). The HABIB catheter can be connected to different RFA generators, among which the most frequently used are the ERBE electrosurgical generators (Erbe,Tübingen, Germany). The recommended settings are effect 8 and 10 Watts (W) for ablation in the common bile duct and 8W for ablating strictures at the biliary bifurcation, near the cystic duct and the ampulla (38, 39). The ELRA catheter (STARmed, Goyang, Korea) is a 7-French temperature-controlled bipolar probe, 175 cm long, with two to four circumferential electrodes in its distal tip which are placed at different lengths. There are four distinct types of ELRA probes which accomplish diverse coagulation lengths (11, 18, 22, and 33 mm) along with circumferential ablation depths between 6 and 8 mm and median ablation depth of 4.0 mm (39, 40, 43, 45). The ELRA catheter operates only with the VIVA comboTM RFA generator (Taewoong Medical, South Korea), which allows presetting the target temperature and automatically stopping the procedure if this is exceeded during the ablation time (45). The recommended settings are a target temperature of 80°C and a power of 7 W or 10 W, on the basis of the type of catheter used as well as the location of the biliary stricture (39, 40, 45). The ELRA catheter differs from the Habib probe due to its different length and its temperature sensor on the tip of the device which provides a temperature-controlled ablation. Theoretically, these features offer the advantage to properly treat biliary strictures of different lengths and to reduce the risks of injuring the biliary ducts (45, 46).

**Table 2:** ERFA Bipolar Catheters (modified from 9).

	HABIB Bipolar Catheter	ELRA Bipolar Catheter
Manufacturer	- Boston Scientific, Marlborough, MA, USA	- STARmed, Goyang, Korea
Diameter/Length	- 8-French (2.6 cm)/180 cm	- 7-French (2.31 cm)/175 cm
Distal Tip	- 24 mm long - two circumferential bipolar electrodes placed 8-mm apart	- 11 or 22 mm long - two circumferential bipolar electrodes - 18 or 33 mm long - four circumferential bipolar electrodes
Median Ablation Depth	- 4 mm	- 4 mm
Ablation Length	- 20-25 mm	- From 11 to 33 mm, depending on the type of probe used
RFA generator	- different RFA generators: - preferred VIO300D electrosurgical generator (Erbe,Tübingen, Germany)	- Only VIVA comboTM RFA generator (Taewoong Medical, South Korea)
Settings	- Power: - effect 8 and 10 W for common bile duct - effect 8 and 8 W for hilum, and ampulla	- Target temperature of 80°C - Power of 7 W or 10 W - type of catheter used - location of the biliary stricture
Energy Control	- YES	- YES
Temperature Control	- NO	- YES
Alarm, if insufficient electrode contact	- NO	- YES

ERFA, Endobiliary Radiofrequency Ablation.

### Procedure

ERFA can be performed at the time of ERCP or PTC before biliary drainage in patients with MBO and strictures due to different neoplastic etiologies (11, 47, 48). Both approaches require cholangiography to properly visualize and measure both the length and the caliber of the stricture, before placing the wire-guided ERFA catheter inside it, under fluoroscopic monitoring (41, 45). The tip of the probe with the electrodes has to be positioned in direct contact with the target tissue. This is crucial for tissue destruction using either one of the devices, with a linear relationship between depth of ablation, preset power and established time of ERFA delivery (40, 49). Usually, each ablation lasts 60-120 seconds, with an average time of 90 seconds (38–41). In the case of long strictures (> 15 mm) the ablation needs to be repeated, without overlapping the treated segments when using the HABIB catheter; on the other hand a different length of the ELRA catheter can be choosen, avoiding repeated ablations for strictures up to 30 mm in length (41, 45). However, two or more ablations are always required when there is a complex hilar stricture, which requires separate treatment of both the right and left hepatic ducts (47). When ERFA is repeated, a 60 s resting period is recommended between applications. After removing the ERFA catheter, the bile duct is swept by using a retrieval balloon to remove residual necrotic tissue and a cholangiogram is obtained to rule out possible complications (24). Eventually, a biliary stent is placed to ensure long term biliary drainage, because of the stricture and the possibility of additional segmental biliary stenosis that ERFA can cause (40). Plastic stents are indicated if periodic ERFA sessions are planned at regular intervals, while metal stents are placed only when a single ERFA is forecasted and performed at the time of biliary drainage (50).

### Technical issues

The therapeutic efficacy of ERFA could be affected by the morphology of the biliary stricture and its location (11, 47). Since the electrodes of the ERFA catheter need to be in strict contact with the target tissue, some features of the biliary stricture can affect the results of the ablation. The narrower the stricture, the higher the amount of energy delivered inside the stenosis: a powerful ablation not only causes deep and irregular coagulative necrosis, but also results in ineffective tissue ablation and increased risk of injury to the duct (18, 51). Similarly, when the biliary stricture is short, irregular or mushy and loose, ERFA could be unsuccessful because of uneven contact between the electrodes of the probe and the target tissue (22, 47, 52). Furthermore, the electrodes of the ERFA catheter can overlap the stricture, and ablate the normal bile duct beyond the biliary stenosis. This usually happens during the ablation of short strictures, but it can also occur with long stenosis when overlapping consecutive ablations are performed (22, 47, 52). In both cases ablation of the normal bile duct develops scar tissue, which expands the length of the original stricture and increases the risk of stent occlusion (40).

Anatomic characteristics of the intrahepatic and hilar biliary ducts might affect the efficacy of ERFA and/or increase the risk of complications. Bile ducts angulation in the hilum can impair ERFA treatment because straight and rigid endobliary catheters may not pass the angulations and/or the tip of the probe may not mantain the required tight contact with the target tissue (53). Hilar and intrahepatic biliary ducts have a subtle wall that is more susceptible to thermal damage, which can extend to surrounding structures, even in the presence of a tumor mass (42, 54). Strictures located in the hilum are close to both portal and hepatic veins as well as hepatic arteries: the blood flow acts as a cooling circuitry (heat-sink effect), which may prevent the ERFA catheter to deliver the proper energy needed to obtain effective tissue ablation (11, 49, 53). Intrahepatic strictures may be difficult to ablate completely because the ERFA catheter cannot pass easily them or the stenoses are too numerous to be all treated effectively (53). In these cases, selective ablation of dominant strictures is performed because complete treatment is not feasible (54).

Several possible solutions to the above mentioned technical issues have been proposed. A preoperative road map with abdominal MRI (magnetic resonance imaging) and MRCP (magnetic resonance cholangiopancreatography) is recommended to accurately assess the tumor surroundings and evaluate the relationship of the target biliary stricture with the surrounding vascular and biliary structures, especially for the treatment of hilar and intrahepatic stenosis (50). Before ERFA, both the length of the stricture and the thickness of the biliary wall should be measured by using intraductal or endoscopic ultrasonography, especially if there is no apparent tumor mass on preoperative imaging (18, 55, 56). This information maximizes the efficacy of ERFA, reducing the risk of thermal injury by means of proper settings of the RFA generator and the duration of ablation, respectively (38–42, 45, 49). Patient-tailored settings may achieve better clinical outcomes for ERFA, which can be ultimately adapted to the native anatomy and the tumor mass (39). The temperature reached by the target tissue during ERFA correlates with the thermal damage of the bile ducts. Therefore, the novel temperature-controlled ERFA system could avoid unintended thermal injury of the biliary wall and the surrounding structures (45, 46). ERFA usually lasts 90-120 seconds. During this time the position of the electrodes may change inside the stricture provoking unintended thermal injury to the normal biliary wall. Therefore, it has been proposed to perform two consecutive 60 seconds ablations with an interval time of 60 seconds which is useful for checking the position of the electrodes by means of fluoroscopy and detecting the possible onset of adverse events, such as bleeding (53). The best way to correctly place the electrodes of the ERFA catheter inside the biliary stricture is to position the probe after direct visualization and evaluation of the stenosis using the peroral digital cholangioscope (57–59). Subsequently, another cholangioscopy evaluates the efficacy of the ablation and rules out possible immediate complications, such as bleeding and perforation (60). Placement of metal stents has been recommended to prevent bile duct injury, because they assure an immediate decompression of the biliary tree and a cooling effect on the ablated tissue by means of a copious biliary flow (23). Placement of fully covered SEMS has been suggested to avoid septic consequences of inadvertent bile duct injury (61, 62).A different technique can be considered for the local treatment of the biliary strictures if the risk of collateral damage induced by ERFA is classified as too high, at the time of preoperative road map (18).

### Adverse events

The major advantages of ERFA are simplicity and low cost, without many major adverse events and very few controindications. The latter include the presence of cardiac devices, coagulation disorders, and ascites, as well as pregnancy (11, 63). There is considerable variability in the reported incidence of the adverse events after ERFA that can range from 7% to 48%, averaging the data of four previous published reviews (42, 56, 63, 64). This variability can be due to the fact that some adverse events after ERFA are not strictly related to it, but are the possible complications after ERCP or PTC, and therefore they might not have been reported (47). Other explanations reside in different etiology, location and morphology of the stricture; degree of thickness of the bile duct wall; contiguity of vascular structures with the biliary stenosis; variance in energy settings and duration of ablation; type of biliary stents (plastic or metal) placed after ERFA (64). The majority of patients complain of abdominal pain, which occurs in almost 50% of cases and it is self limited (10, 14, 65–67). Reported pancreatobiliary adverse events are pancreatitis, cholangitis, cholecystitis and minor bleeding (10, 14–16, 18–20, 22, 23, 44, 46, 50, 55, 61, 65, 66, 68–74). These are the typical adverse events that can occur after ERCP or PTC (5–8, 75). However, a higher number of cholecystitis and cholangitis have been reported, especially in patients with hilar strictures treated with ERFA (10, 22, 23, 50, 65, 68, 69, 73, 76). The incidence of cholecystitis requiring percutaneous drainage after ERFA has been estimated to be between 2%–4% and it is significantly higher than that reported after standard biliary drianage; similarly, cholangitis seems to occur more frequently after ERFA and subsequent placement of biliary stents (2-8%) (24). A possible explanation for the onset of suppurative cholecystitis could be the obstruction of the cystic duct, as a consequence of its thermal injury due to edema or tissue destruction when ERFA is delivered too close to the opening in the bile duct (10, 65). However, cholecysistis is not always reported after ERFA (18), and it has been hypothesized that the type (plastic vs metal) and the number (1-2 vs multiple) of biliary stents could be associated with a higher risk of cystic duct blockage (13). Since the reported total number of cases of cholecystis remains low and this complication resolves in few days with percutaneous gallbladder drainage and/or antibiotics, ERFA with subsequent biliary drainage is considered safe, even when the biliary stricture is close to the opening of the cystic duct (65). The high frequency of cholangitis has been initially attributed to necrotic debris which can remain in the bile ducts after ERFA with early subsequent obstruction of biliary stents; to avoid this possible complication the bile ducts are swept with an extraction ballon after ERFA and before stent placement (24, 48, 65, 77). Another possible cause of cholangitis is the ablation of the normal bile duct beyond the stricture, which sometimes occur because of technical and/or anatomical difficulties determining the onset of iatrogenic strictures of the bile ducts which could not be properly stented (40, 47). To reduce the risk of unintended strictures an accurate measurament of the stricture is required, especially if the biliary stenosis is long and requires ovelapping ERFA (18, 55, 56, 78). Few life-threathening adverse events have been reported (20, 36, 50, 62, 79–81). Therefore, it is important for the biliary endoscopist to be aware of these complications. Seven cases of biliary perforations occurred after endoluminal ablation of narrow biliary strictures, two of which were dilated before performing two overlapping ablations (20, 62, 73). After ERFA late severe melena developed in two patients and this was due to the rupture of a pseudoaneurysm originating from an artery, which was too close to the electrodes of the ablation catheter (79, 81). Six cases of delayed hemobilia were reported 4-6 weeks after ERFA and two of them were fatal because of hemorrhagic shock (41, 50). Liver infarction due to arterial thrombosis was diagnosed in a patient 3 days after ERFA of a stricture of the right hepatic duct: this complication was attributed to the proximity of the biliary stricture with a branch of the right hepatic artery (50). Vascular as well as biliary injuries ending in severe complications are mostly related to severe thermal injury of the bile duct inside and beyond the stricture which extends to the surrounding vascular and biliary stricture (50, 79, 81). Furthermore, aberrant angiogenesis after ERFA could explain delayed spontaneous hemobilia (41, 50). Placement of a SEMS after ERFA could be an effective method for preventing the onset of late bleeding and biliary fistulas. It has been hypothesized that the high radial force of SEMS may have both a tamponade and hemostatic effect on the oozing from the necrotic tissue resulting after ERFA (25, 78). The rapid flow of bile through the strictures ensured by SEMS could have a cooling effect preventing deep bile duct injuries (23). Hyperkalemia was the cause of a sudden non-fatal cardiac failure in a patient with chronic kidney disease who underwent ERFA for the treatment of a biliary stricture at the time of biliary drainage (80). Another three cases of non-lethal heart failure occurred in two patients with a history of coronary heart disease and hypertension within 24 hours after ERFA (20, 74). Finally, a case of fatal hepatic coma, a left bundle branch block, and a few cases of liver abscess have been reported, especially after ERFA at the time of PTC (16, 19, 21, 22, 50, 54).

Strict patient-selection and ablation with customized settings (according to the location of the biliary stricture and the comorbidity of the patient) have been proposed to reduce the incidence of severe complications (20). Careful postoperative follow-up is necessary, and evaluation of the results of ERFA with cholangioscopy has been recommended (47, 60, 66).

## Beneficial effects of ERFA in the care of unresctable cholangiocarcinoma

Patients with unresectable CCA have an overall survival of approximately 10 months if they undergo chemotherapy and about 4 months if they receive best supportive care (BSC) (3). The most common regimen of chemotherapy is based on the association between gemcitabine and cisplatin, which significantly reduces the risk of death compared to BSC or gemcitabine alone (1–3). In case of failure, modified FOLFOX should be used as second-line treatment, with a median progression free survival and median overall survival of 3.2 and 7.2 months, respectively (1, 2). Recently, a subset of patients showing isocitrate dehydrogenase isoenzyme 1 mutations (mIDH1) had been treated with ivosedinib, an oral small molecule inhibitor of mIDH1 with a median progression free survival of 2.7 months (2). Despite all these efforts, the prognosis of patients with unresctable CCA undergoing chemotherapy remains dismal (3, 4).

Among endobiliary therapies ERFA is the best option for its semplicity, low cost and relatively few serious side effects (24, 25, 31). At the time of biliary drainage of jaundiced patients with unresectable CCA, ERFA could be used as adjuvant therapy with the aim to control the biliary and peribiliary growth of the tumor (24). The majority of published studies have mainly evaluated the role of ERFA in the management of biliary obstruction due to bilio-pancreatic cancer, considering its impact on both stent patency and overall survival of the patients (10, 14, 17, 19–22, 37, 39, 41, 45, 46, 50, 51, 57, 66, 69, 71–73, 76, 78). The hypothetical beneficial effects of ERFA on palliative treatment of unresectable CCA has been investigated in the recent years (15, 16, 18, 23, 44, 52–55, 61, 67, 68, 70, 74). Three are single arm studies aimed to mainly assess both feasibility and safety of ERFA (**Table 3**) (44, 61, 74). Eight comparative studies, three of whom were randomized controlled trials (**Table 4**) and five were retrospective studies (**Table 5**), explored the impact of ERFA on stent patency, overall survival and improved functional status of the patients (15, 18, 23, 53, 54, 67, 68, 70). Finally, three studies evaluated the hypothesis that the combination between ERFA and chemotherapy could have a cumulative beneficial effect improving the overall survival as well as the quality of life in patients with locally advanced unresectable CCA (16, 52, 55). Recently, a meta-analysis evaluated the results of nine comparative studies, which had assessed both stent patency and overall survival in patients with unresectable CCA undergoing ERFA (65). The majority of these studies reached the conclusion that ERFA improves both stent patency and overall survival of patients with unresctable CCA. However, it is still unclear if these beneficial effects are related or independent, since the improved overall survival could be the consequence of prolonged stent patency.

**Table 3:** Single arm studies evaluating feasibility and safety of ERFA for NR-CCA (modified from 47).

Study	Study Design	#Patients	Localization and Bismuth Classification of NR-CCA	Procedure	# ERFA sessions	Type of biliary stent	Technical Success	Stent Patency median -range	Survival *mean - range median- range	Adverse events
44. Laquière A, et al. Surg Endosc (2015) (44)	Bicentric Case Series	12	Hilar = 12 4 Bismuth I 3 Bismuth II 2 Bismuth III 3 Bismuth IV	ERCP	19 (1-3 sessions) 5 pts = 2 sessions 1 pts = 3 sessions	Plastic	100%	NR	*12.3 mos (3-31)	1 cholangitis 1 Sepsis No mortality
62. Alis H, et al. Hepatobiliary Pancreat Dis Int (2013) (61)	Retrospective Single Arm	10	Distal = 6 Hilar = 4 4 Bismuth I	ERCP	10 1 session per pts	Metal = FC-SEMS	90%	9 mos (6-15)	NR	2 pancreatitis No mortality
76. Wang Y, et al. Oncotarget (2016) (74)	Retrospective Single Arm	9	Hilar = 9 2 Bismuth III 7 Bismuth IV	PTC	10 9 pts = 1 session 1 pts = 2 sessions	Metal = U-SEMS	100%	100 days (85-115)	5.3 mos (2.5-8.1)	3 abdominal pain 4 cholangitis 1 atrial fibrillation No mortality

In all the studies ERFA was performed using the HABIB bipolar probe with the following setting of RFA generator: 10 Watts.

NR-CCA, non resectable cholangiocarcinoma; ERFA, endobiliary radiofrequency ablation; NR, not reported; mos, months; pts, patients; U-SEMS, uncovered self expanding metal stent; FC-SEMS, fully covered self expanding metal stents.

**Table 4:** Comparative randomized controlled studies evaluating the impact of ERFA on the management of NR-CCA (modified from 9, 47).

Study	#Patients		Localization and Bismuth Classification of NR-CCA		Proce-dure	ERFA Probe	RFA generator settings	# ERFA sessions	Type of biliary stent	Stent Patency Time median-mean range			Overall Survival Time median-mean range			Adverse Events (%) median-mean		
	ERFA + Stent	Stent	ERFA + Stent	Stent						ERFA + Stent	Stent	P	ERFA + Stent	Stent	P	ERFA + Stent	Stent	P
18. Yang J, et al. Endoscopy (2018) (18)	32	33	Distal = 22 Hilar = 10 Bismuth I-II	Distal = 24 Hilar = 9 Bismuth I-II	ERCP	HABIB	7-10 W	Repeated ERFA, every 6 months depending on IDUS results	Plastic	6.8 mos 3.6 -8.2	3.4 mos 2.4 -6.5	0.02*	13.2 mos 11.8-14.2	8.3 mos 7.3-9.3	<0.001*	6%	9%	>0.05
54. Kang H, et al. J Hepatobiliary Pancreat Sci (2022) (54)	15	15	Hilar = 15 2 Bismuth II 6 Bismuth III 7 Bismuth IV	Hilar = 15 3 Bismuth II 8 Bismuth III 4 Bismuth IV	ERCP	ELRA	7 W T = 80°C	Repeated ERFA and replacement of plastic stent with U-SEMS after 3 months	Plastic and then Metal = U-SEMS scheduled stent exchange at 3 months	178 days 96-260	122 days 111-139	0.154	230 days 77-383	144 days 0-323	0.643	60%	73%	>0.05
70. Gao D-J, et al. Gastrointest Endosc (2021) (68)	87	87	Distal = 62 (including ampullary cancer) Hilar = 25 8 Bismuth I 9 Bismuth II 8 Bismuth III	Distal = 65 (including ampullary cancer) Hilar = 22 10 Bismuth I 7 Bismuth II 5 Bismuth III	ERCP	HABIB	7-10 W	Repeated ERFA at scheduled stent exchange every 3 months	Plastic	3.7 mos	4.1 mos	0.674	14.3 mos	9.2 mos	<0.001*	28%	19%	0.21

NR-CCA, non resectable cholangiocarcinoma; ERFA, endobiliary radiofrequency ablation; IDUS, intraductal ultrasonography; NR, not reported; mos, months; U-SEMS, uncovered self expanding metal stent.

*statistically significant.

**Table 5:** Comparative Retrospective Studies evaluating the impact of ERFA on the management of NR-CCA (modified from 9, 47).

Study	#Patients		Localization and Bismuth Classification of NR-CCA		Proce-dure	ERFA Probe	RFA generator settings	# ERFA sessions	Type of biliary stent	Stent Patency Time median-mean range			Overall Survival Time median-mean range			Adverse Events (%) median-mean		
	ERFA + Stent	Stent	ERFA + Stent	Stent						ERFA + Stent	Stent	P	ERFA + Stent	Stent	P	ERFA + Stent	Stent	P
15. Bokemeyer A, et al. Scientific Reports (2019) (15)	20	22	Hilar = 20 1 Bismuth III 19 Bismuth IV	Hilar = 22 2 Bismuth I 20 Bismuth II	ERCP	HABIB	8 Watts (22 sessions) 10 Watts (19 sessions) Others (5 sessions)	Repeated Sessions in 40.7% of cases	Plastic (85% vs 91%) Metal = U-SEMS(15% vs 9%)	NR	NR	–	342 days	221 days	0.046*	18.5%	NR	–
23. Liang H, et al. Journal of Cancer Therapy (2015) (23)	34	42	Distal = 22 Hilar = 12 All Bismuth I	Distal = 27 Hilar = 15 All Bismuth I	ERCP (29 vs 37) PTC (5 vs 5)	HABIB	10 Watts	Repeated Sessions in 11.8% of cases	Metal = U-SEMS (30 vs 36) FC-SEMS (4 vs 6)	9.5 mos 4.5-14	8.3 mos 4.9-11	0.024*	12.8	11.3	0.036*	26.5%	23.8%	>0.05
53 Oh D, et al. Journal of Gastroenterol and Hepatology (2022) (53)	28	51	Hilar = 26 1 Bismuth I 2 Bismuth II (GB cancer) 14 Bismuth III 11 Bismuth IV	Hilar = 36 1 Bismuth I 9 Bismuth II (GB cancer) 19 Bismuth III 22 Bismuth IV	ERCP	ELRA	7–10 W T = 80°C	NO	Metal = U-SEMS	192 days	140 days	0.41	311 days	311 days	0.73	7.1% (early)	2% (early)	0.25
69. Wu TT, et al. Cardiovasc Intervent Radiol (2017) (67)	35	36	Distal = 35	Distal = 36	PTC	HABIB	10 Watts	No	Metal = U-SEMS = 58 FC-SEMS = 13	241 days (28 U-SEMS)	137 days (30 U-SEMS)	0.001*	245 Days (28 U-SEMS	209 days (30 U-SEMS	>0.05	0% severe events (28 U-SEMS)	16.6% severe events (30 U-SEMS)	–
72. Qi S, et al. Am J Transl Res (2021) (70)	60	60	NR	NR	PTC	NR	NR	No	Metal = U-SEMS	NR	NR	–	11.1 mos	8.2 mos	<0.001*	15%	25%	0.114

NR-CCA, non resectable cholangiocarcinoma; ERFA, endobiliary radiofrequency ablation; NR, not reported; U-SEMS, uncovered self expanding metal stent; FC-SEMS, fully covered self expanding metal stent.

*statistically significant.

### ERFA and stent patency

Maintaining the patency of biliary stents guarantees the administration of chemotherapy without interruption. Despite the use of biliary SEMS, recurrent jaundice and cholangitis tend to occur because of reobstruction of the biliary stents due to tumor growth (7, 8). Several studies have demonstrated the beneficial effects of ERFA on stent patency, which is usually prolonged by approximately 2 months (20–23). The effects of ERFA on stent patency has been investigated by the majority of the cited comparative studies (15, 18, 23, 53, 54, 67, 68, 70). It seems that ERFA has the capability to prolong the patency of uncovered metal stents inducing a reduction in the tumor mass, which is associated with slowed endobiliary neoplastic growth and improved bile flow (52, 54). The decreased risk of sludge and/or biofilm formation could also explain the prolonged patency of plastic stents fter ERFA (54). As mentioned above, three single arm studies confirmed that ERFA can be performed safely at the time of biliary drainage either by means of ERCP or PTC and followed by placement of plastic or metal stents (44, 61, 74). The advantage of using plastic stents is that they permit repeated sessions of ERFA at scheduled times and this protocol seems to be beneficial for patients with unresectable CCA (15, 18, 44, 68). The impact of ERFA on biliary stent patency has been confirmed by a recent meta-analysis whose data demonstrated the superiority of ERFA plus stenting over stenting alone, independent of the stent type used (plastic vs metal) (10). However, these data are still controversial since it has been reported that ERFA has no effect on prolonging patency of both metal and plastic stents, respectively by a retrospective study and two randomized controlled trials (53, 54, 69). Similar doubtful and inconclusive results were obtained by a recent meta-analysis whose authors were unable to perform a pooled analysis of avalaible data and just reported that only three of five studies evaluated showed a beneficial impact of ERFA on stent patency (65). Biliary plastic stents need to be exchanged and this can be performed respectively, on schedule every three months or on demand (i.e. at the occurrence of signs and/or symptoms of obstruction) after the second session of ERFA scheduled at the time of first 3-month endoscopic follow-up (18, 68). When plastic stents are exchanged, a repeat ERFA session can be performed. The need of reintervention could be decided on the basis of the results of cholangiography and/or intraductal ultrasonography (IDUS), which can measure the caliber and the width of the bile duct (18, 48, 56, 66). ERFA should be repeated when IDUS detects a significant increase in tumor thickness and a reduction in the bile duct diameter at the site of the previously treated stricture (18). Another technique used to monitor the results of ERFA is cholangioscopy which can also guide the correct placement of the ERFA catheter inside the targeted biliary stricure (58–60). Ideally, plastic stents are indicated when multiple sessions of ERFA are scheduled in patients with a locally advanced CCA without metastases and in good functional status (24). On the other hand, SEMS are recommended when only a single session of ERFA is planned and their use has been advocated to reduce the risk of late bleeding and biliary fistulas (23, 25, 78). Moreover, SEMS are the preferred stents after the execution of ERFA at the time of PTC, which is usually performed to treat intrahepatic unresctable CCA (23, 48, 65, 67, 70).

### Survival benefit of ERFA

The most valuable effect of ERFA is its impact on the overall survival of patients with unresectable CCA undergoing biliary drainage and stent placement. The above mentioned comparative studies as well as the cited meta-analysis investigated the impact of ERFA on overall survival and improved functional status of patients (15, 18, 23, 53, 54, 65, 68, 70). The overall survival of patients with unresectable CCA is significantly improved after ERFA plus stenting, with a pooled mean survival of 374 days vs 263 days of those treated only with stent placement at the time of biliary drainage (15, 18, 23, 68, 70). Similar data were obtained by the meta-analysis which reported a median survival of 294 days in patients undergoing ERFA vs 216 days in those who received only a biliary stent, independent from the type of stent placed (65). As already mentioned, the improved survival of patients undergoing ERFA could be due to the local immunogenic response, which potentially enhances tumor suppression and decreases the tumor burden delaying neoplastic progression inside as well as outside the bile duct (20–23). The direct action on the tumor and the induced local and systemic immune mechanisms could explain the favorable impact of ERFA on overall survival of patients undergoing endobliary ablation (65, 68). Only two retrospective studies reported no difference in overall survival between patients undergoing ERFA and those treated only with biliary stent placement (53, 54). There are several possible explanations for these controversial results: the anatomy of the biliary ducts, which could have been too angulated for adequate ablation; the cooling effect due to the blood flow of the surroundings vessels which could have prevented sufficient ablation of the tumor; the type of CCA treated, since Bismuth III and IV are characterized by the presence of multiple strictures, which could not be ablated as a whole, invalidating the efficacy of ERFA; the placement of SEMS which could have hidden the beneficial effects of ERFA; the use of different probes and generator settings which could have affected the outcomes, especially in patients with hilar CCA undergoing ERFA (53, 54). Besides overall survival, ERFA seems to have also a beneficial effect on the functional status of the patients undergoing endobiliary ablation. Several studies reported rapid improvement of the jaundice and increased albumin values which translated to a better functional status and higher Karnofsky Performance Scale (KPS) scores in comparison with patients treated only with stent placement (18, 23, 68, 70). ERFA, cancer stage, Bismuth type I-III, level of serum albumin near normal and the administration of adjuvant chemotherapy could be positive prognostic factors that have a beneficial cumulative impact on the overall survival of patients with unresectable CCA (23, 65, 68). Among these, adjuvant chemotherapy has been proven to be the most effective and its combination with ERFA could be the best option to improve the overall survival of patients with advanced CCA (65).

### Impact of the combination of ERFA and adjuvant chemotherapy on overall survival

It has been postulated that the thermal cell injury induced by ERFA could increase the cytotoxic effect of chemotherapy, especially in the case of intrahepatic CCA where the endobiliary ablation is often sublethal (53). Moreover, some data suggest that stent patency is shorter after ERFA without chemotherapy, and failure of its administration can be considered a risk factor for stent occlusion in patients with unresectable CCA undergoing endobiliary ablation (23, 53). The possible advantage of the combination between ERFA and adjuvant chemotherapy has been investigated in three studies, two of which were retrospective and the other one was a randomized controlled trial (16, 52, 55). In the two retrospective studies patients undergoing combination therapy were compared with those treated only with chemotherapy after biliary drainage with the aim of evaluating the impact of combination therapy on both the overall survival and the progression free survival of patients with unresectable CCA (**Table 6**) (16, 52). The superiority of combination therapy over ERFA alone was then demonstrated by a randomized controlled trial which investigated the effect of the consecutive administration of ERFA and a novel anti-cancer drug in improving both overall survival and progression free survival of patients with locally advanced unresctable CCA (55). All the data presented in these studies support the efficacy of the additional effect of ERFA on chemotherapy, with an average median survival of 16.6 months compared to 10.3 months of patients undergoing only chemotherapy (16, 52, 55). Similarly, median progression free survival (PFS) was improved in patients undergoing chemotherapy after ERFA (16, 52). These advantages were clear for locally advanced CCA, but became less evident in patients with metastatic CCA, for whom the combination of ERFA and chemotherapy did not significantly increase both median survival and PFS in comparison to patients undergoing chemotherapy alone (16, 52). The combined therapy also had a beneficial impact on the functional status and the quality of life of patients with a prolonged high KPS scores after ERFA (55). No major side effects of both treatments and no increase in adverse events were reported with the combination of ERFA together with adjuvant chemotherapy (16, 52, 55). Therefore, the results of these three studies support our change of approach in patients with locally advanced CCA undergoing biliary drainage, especially if they have a life expectancy of at least 6 months: if possible, they should undergo ERFA before starting adjuvant chemotherapy (82).

**Table 6:** Comparative studies evaluating the impact of ERFA plus Chemotherapy on the management of NR-CCA (modified from 9, 47).

Study	Design	#Patients		Localization and Bismuth Classification of NR-CCA		Proce- dure	ERFA Probe	RFA generator settings	# ERFA sessions	Type of biliary stent	Overall Survival Time median-mean			Progression Free Survival			Adverse Events (%) median-mean		
		ERFA + Stent + CHT	Stent + CHT	ERFA + Stent + CHT	Stent + CHT						ERFA + Stent + CHT	Stent + CHT	P	ERFA + Stent + CHT	Stent + CHT	P	ERFA + Stent + CHT	Stent + CHT	P
16 Gonzalez− Carmona MA et al. Scientific Reports (2022) (16)	Retrospective	40	26	Distal + Hilar Bismuth I-II = 9 Hilar Bismuth III-IV = 31	Distal + Hilar Bismuth I-II = 7 Hilar Bismuth III-IV = 19	ERCP	HABIB	10 Watts	Repeated Sessions in 55% of cases	Plastic Metal in case of early disfun-ction	17.3 mos	8.6 mos	0.004*	12.9 mos	5.7 mos	0.045*	72.5% Cholangitis Similar Hematologic toxic effects	53.8% Cholangitis Similar Hematologic toxic effects	0.031*
52. Inoue T, et al. Curr Oncol (2022) (52)	Retrospective	25	25	Distal = 4 Hilar = 21	Distal = 3 Hilar = 22	ERCP	HABIB	7-10 Watts	1 session	U-SEMS	17.1 mos	11.3 mos	0.017*	8.6 mos	5.8 mos	0.014*	8% 1 Cholangitis 1 Pancreatitis Similar Hematologic toxic effects	8% 1 Cholangitis 1 Bleeding Similar Hematologic toxic effects	1.000

NR-CCA, non resectable cholangiocarcinoma; ERFA, endobiliary radiofrequency ablation; NR, not reported; U-SEMS, uncovered self expanding metal stent; FC-SEMS, fully covered self expanding metal stent.

*statistically significant.

## Conclusions

**Figure 2:**
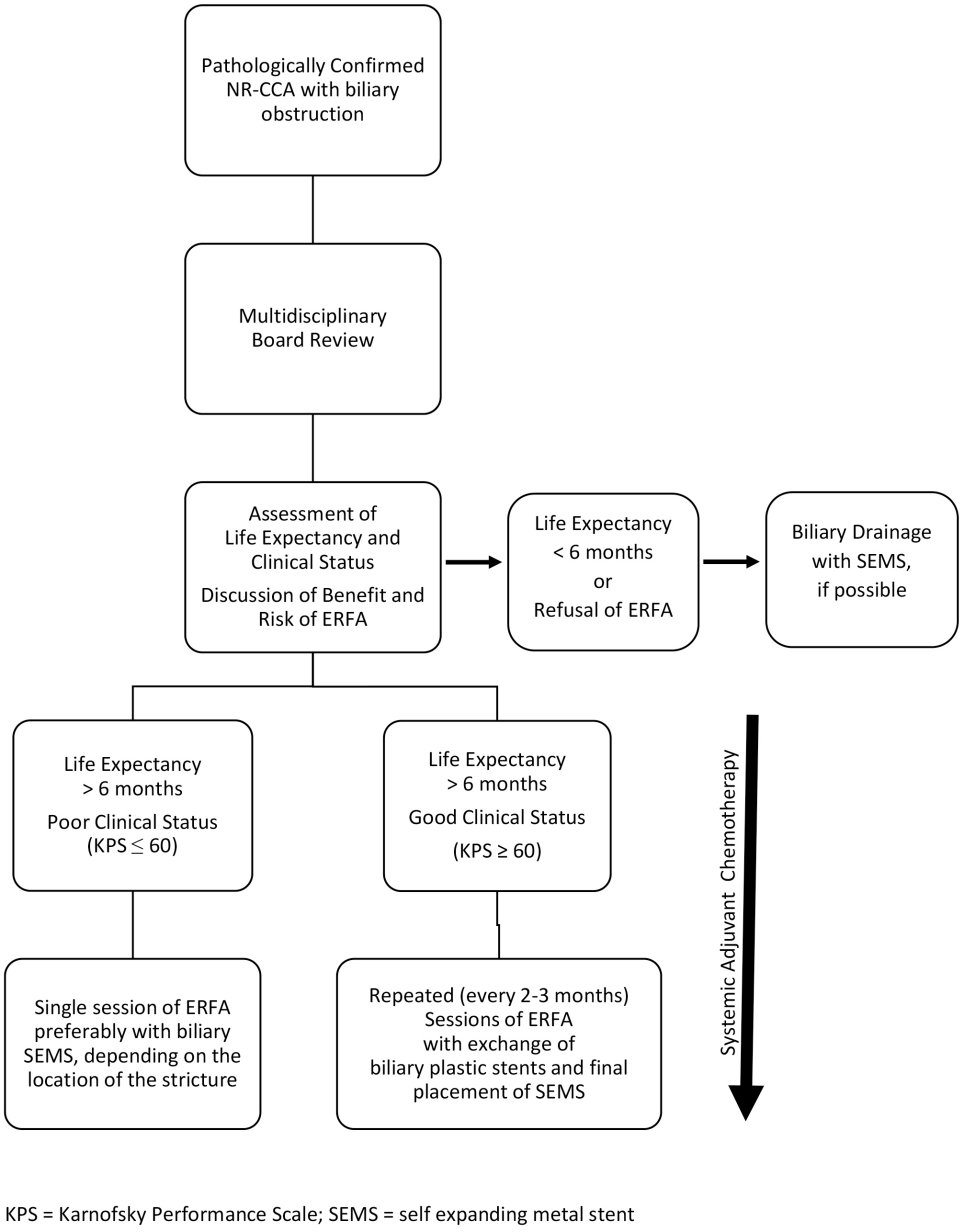
Proposed algorithm for Endobiliary Radiofrequency Ablation (ERFA) in the management of jaundiced patients with locally advanced unresectable cholangiocarcinoma (NR-CCA), Bismuth type I-III (modified from 8 and 23).

Available literature data support the role of ERFA as adjuvant therapy which increases both stent patency and overall survival in patients with unresectable CCA. These beneficial effects could add up to those of chemotherapy, with a cumulative impact of combination therapy on functional status, PFS and overall survival of patients with unresectable CCA. In the light of its potential benefit, ERFA could become part of the management of patients with locally advanced unresectable CCA, Bismuth type I-III, with a prognosis of at least 6 months (**Figure 2**) (83). However, there are still some issues that need to be clarified regarding the settings of the RFA generator, the type of ERFA catheter (energy and temperature controlled vs energy controlled), the number of ablations, the frequency of sessions (if more than one), and the type of biliary stent (metal vs plastic) in order to develop a standardized protocol. All these questions require further research and some of them could be answered in the three ongoing clinical trials which are investigating the role of ERFA for the treatment of unresectable CCA (84–86).

## Author contributions

EDG and MDB managed the overall project and prepared the first draft. LT, FF, AO and AB reviewed the first draft. All other authors reviewed the manuscript and contributed to the final version of the article. EDG and MDB finalized the article on the basis of comments from the other authors. All authors contributed to the article and approved the submitted version.
